# Hydrophobic Fibers with Hydrophilic Domains for Enhanced Fog Water Harvesting

**DOI:** 10.3390/polym18030425

**Published:** 2026-02-06

**Authors:** Joanna Knapczyk-Korczak, Katarzyna Marszalik, Marcin Gajek, Urszula Stachewicz

**Affiliations:** 1 Faculty of Metals Engineering and Industrial Computer Science, AGH University of Krakow, al. A. Mickiewicza 30, 30-059 Krakow, Poland; jknapczyk@agh.edu.pl (J.K.-K.); marszalik@agh.edu.pl (K.M.); 2 Faculty of Materials Science and Ceramics, AGH University of Krakow, al. A. Mickiewicza 30, 30-059 Krakow, Poland; mgajek@agh.edu.pl

**Keywords:** electrospinning, fibers, polyurethane, cellulose acetate, hydrophilic–hydrophobic, fog water collection

## Abstract

Fog water collectors (FWCs) present a sustainable solution for arid regions where fog is a primary water source. To improve their efficiency, we developed a durable and high-performance mesh composed of electrospun hydrophobic thermoplastic polyurethane (TPU) fibers combined with hydrophilic cellulose acetate (CA) microbeads. This hybrid design represents a novel biomimetic strategy, mimicking natural fog-harvesting mechanisms by optimizing wetting and drainage. Despite the significant reduction in average fiber diameter, the TPU-CA mesh maintained mechanical strength close to 1 MPa, comparable to pristine TPU. The introduction of hydrophilic domains into a hydrophobic fibrous network is a unique architectural approach that enhanced fog collection performance, achieving a high water harvesting rate of 127 ± 12 mg·cm^−2^·h^−1^. Remarkably, although the mesh remained predominantly hydrophobic, droplets shed completely from its vertical surface, exhibiting near-zero contact angle hysteresis. This synergistic wetting concept enables performance unattainable with conventional single-wettability meshes. Compared to single-material meshes, the TPU-CA hybrid showed nearly double the water collection efficiency. The innovative interplay between surface chemistry, microscale heterogeneity, and mechanical robustness is key to maximizing water capture and transport, offering a promising path for scalable, efficient FWCs in poor water-stressed regions.

## 1. Introduction

Many water-stressed regions lack reliable access to freshwater, yet often experience frequent fog events, making the efficient capture of fog water a key challenge for sustainable supply. Researchers developed fog water collectors (FWCs) by mimicking natural systems to improve living standards in the arid regions [1,2,3]. FWCs are utilized to supply water to regions where access to conventional water resources is severely constrained. Using specially designed mesh structures mounted on steel stands, FWCs can capture water from passing fog [4,5,6]. For commonly used constructions the water collection rates range from 3 to 10 L/m^2^ per day [7]. The efficiency of water harvesting is influenced by environmental factors such as wind speed, fog density, and the water content dispersed within the fog [8,9]. Maintaining permeability and preventing pore blockage is critical to secure the high water collection. For instance, the commercial Raschel mesh, with a shade coefficient of 35%, is mounted in a double-layer configuration, providing an open area for wind passage of approximately 40% [5]. Ongoing modifications to meshes aim to prevent pore blockage caused by accumulated droplets and mitigate the wind-induced re-entrainment of water collected on ribbons [10].

Climate change has increased the frequency of droughts worldwide [11], requiring innovative ways to harvest water under harsh hydrological conditions, often inspired by natural systems [12,13]. Biomimicry offers technological solutions by emulating strategies evolved in animals and plants [14,15]. Many water-collection strategies mimic organisms from arid regions, such as Namib desert beetles (*Stenocara gracilipes*, *Onymacris unguicularis*) [16,17], the thorny devil lizard (*Moloch horridus*) [18,19], cacti (*Opuntia microdasys, Copiapoa cinerea*) [20,21,22], and desert grass (*Stipagrostis sabulicola*, *Setaria viridis*) [23,24]. For example, the Namib desert beetle has a hydrophobic exoskeleton with hydrophilic bumps that collect water droplets from moist air, while the surrounding hydrophobic areas channel droplets efficiently toward the beetle’s mouth [25,26]. Some desert and mountain spider species construct webs that promote condensation of fog or dew on the silk threads, allowing the spiders to drink the collected droplets [27]. One of the methods to produce highly porous meshes able to catch water from the air is electrospinning [28]. It is a versatile technique for producing micro- and nanofibers from polymer solutions [29,30]. The electrostatic forces used in this process allow the surface tension of the solution to be overcome and generate a continuously charged polymer jet [31,32,33]. During the fiber formation, all solvents evaporate, and fibers are collected as a nonwoven mesh on the collector [34,35]. An electrospun mesh is inspired by a spider web, an exceptionally strong and tough material due to spider silk’s unique combination of amino acids [36,37,38,39]. It demonstrates the ability to collect water through the synergistic integration of hydrophobic and hydrophilic parts within its structure, and the appearance of Laplace pressure, which facilitates the accumulation of water on the beads present along the fibers [40,41]. An innovative strategy for improving existing systems involves the integration of nano- and microfibrous structures. For example, the incorporation of polymer nanofibers into Raschel meshes has been reported to substantially enhance their performance, achieving up to a 300% increase in water collection efficiency compared to conventional mesh designs [42,43]. Recent research on nanofibers and yarns focuses on their application in advanced water-harvesting systems, utilizing the combination of hydrophobic and hydrophilic properties to enhance efficiency [44,45,46,47,48]. One approach is to design a system combining wetting properties and incorporating beads to enhance water absorption efficiency [49,50,51]. Fog water harvesting also advances with active systems like electrostatic and photoresponsive materials. Electric fields attract droplets using high voltage, improving efficiency over conventional methods [52,53,54]. Photoresponsive fibers, such as TiO_2_-PVDF meshes, switch wettability under UV light and heat, enhancing water capture in low humidity [47,55].

**Figure 1:**
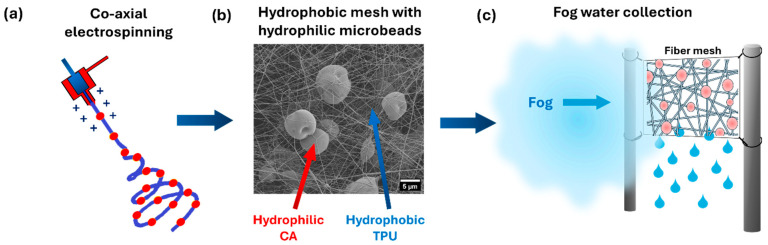
The concept of the work presents the (**a**) co-axial electrospinning of TPU and CA to produce (**b**) an electrospun hydrophobic fiber mesh from TPU with hydrophilic microbeads from CA capable of (**c**) harvesting water from fog.

The aim of this study is to develop a durable, biomimetic mesh capable of efficiently collecting water by integrating hydrophobic electrospun fibers with hydrophilic microbeads to enhance fog harvesting performance. This approach is innovative because it introduces hydrophilic domains into a hydrophobic fibrous mesh, leveraging the contrasting wetting properties to enhance both droplet nucleation and drainage efficiency, thereby maximizing water capture and transport, as shown in Figure 1. The TPU-CA mesh in our study combines wetting properties; however, it differs fundamentally from the Janus structure. Unlike the Janus structure, which has distinct hydrophobic and hydrophilic sides, our TPU-CA mesh is a unified system without such a separation. Additionally, Janus structures often require materials with different geometries and post-treatment steps, which can increase production costs. The TPU-CA system presented here is created in a one-step process.

## 2. Materials and Methods

### 2.1. Materials and Electrospinning

Prior to the solution preparation, the cellulose acetate (CA, M_w_ = 50,000 g·mol^−1^, Sigma-Aldrich, Gillingham, UK) and thermoplastic polyurethane (TPU, Elastollan 1185 A15, BASF, Ludwigshafen, Germany) were dried at T = 30 °C in a laboratory drying machine (Pol-Eko, Wodzisław Śląski, Poland). To prepare polymer solutions, the following solvents were used: acetone, dimethylacetamide (DMAc), dimethylformamide (DMF), tetrahydrofuran (THF), all pure P.A. (ACS) (Avantor, Gliwice, Poland). To prepare pristine fiber meshes, TPU comprising 15 wt% was dissolved in a mixture of THF-DMF, while CA with a concentration of 19 wt% was dissolved in DMAc-acetone, each utilizing a 1:1 volume ratio. Solutions of TPU at 15 wt% dissolved in DMF and CA at 8 wt% in DMAc-acetone were used for co-axial electrospinning. All solutions were stirred for 3–5 h at T = 25 °C at a speed of 150–400 rpm (RCT basic, IKA, Staufen, Germany).

TPU fibers were produced by electrospinning using ESVY-100 (MicroNano Tools, Niagara Falls, ON, Canada). The CA mesh was electrospun in the chamber with humidity control (IME Technologies, Waalre, The Netherlands). TPU and CA fibers were electrospun using a standard stainless-steel nozzle (OD = 0.8 mm, gauge G21, length = 4 cm). To obtain TPU fibers with CA microbeads (TPU-CA) a co-axial nozzle was used (ID = 0.3 mm for core, and ID = 1.3 mm for shell), see Figure S1a in the Supporting Information. The electrospun meshes were deposited onto paper on a rotating drum (10 rpm) for 2 h (TPU and TPU-CA) and 4 h (CA). For mechanical testing, the samples were electrospun directly onto paper frames for 30 min. All used parameters are listed in Table S1 in the Supporting Information.

### 2.2. Materials Characterization

Microstructural analysis of the obtained fibers was performed using a scanning electron microscope (SEM, Merlin Gemini II, ZEISS, Oberkochen, Germany). Before imaging, all samples were coated with 8 nm Au using a rotary pump sputter coater (Q150RS, Quorum Technologies, Laughton, UK). Operating parameters included an accelerating voltage of 2.5 kV, a current of 110 pA, and a working distance of 5–8 mm. Average fiber diameters were determined via ImageJ software (version 1.50i, National Institutes of Health, Bethesda, MD, USA) based on 100 measurements per sample. The diameter of every microbead was measured diagonally at three different points, and the average diameter was calculated from a total of 100 measurements. Fiber and bead size distribution histograms were generated using OriginPro software (2018b, OriginLab, Northampton, MA, USA).

SEM micrographs of the TPU–CA meshes were used to determine the ratio of fiber and bead fractions. Prior to analysis, binary images were generated using the Li thresholding method. Threshold values were set to 60 for fibers with beads and 120 for bead-only regions. In the binary images, white pixels corresponded to the polymer phase and black pixels represented pores, together accounting for 100% of the image area. Based on the distribution of white pixels, the fractions of fibers with beads and of isolated beads were quantified, and the difference between these values was taken as the fiber fraction. All images were processed using the particle analysis function in ImageJ software. Representative binary images are provided in Figure S2d–f in the Supporting Information.

The molecular structure of the obtained TPU and CA fibers, as well as the contents of both polymers in the TPU-CA mesh, were examined using Fourier transform infrared spectroscopy (FTIR, Nicolet iS5, Thermo Fisher Scientific, Waltham, MA, USA). The analysis involved 64 scans utilizing the diamond ATR module (iD7 ATR–Diamond), with a resolution and wavelength range set at 4 cm^−1^ and 400–4000 cm^−1^, respectively. The FTIR peaks were analyzed using the OMNIC 9 software (version 9.12.928, Thermo Fisher Scientific, Waltham, MA, USA), while graphs were generated with OriginPro.

A tensile module equipped with a 20 N load cell (Kammrath Weiss GmbH, Schwerte, Germany) was used to verify the mechanical properties of the obtained meshes. Fibers, electrospun directly onto the paper frame with a 1.8 × 2 mm gap, were mounted between device clamps. The fibers were then uniaxially stretched at an extension rate of 25 μm·s^−1^. At least three measurements were taken for each type of fiber mesh. The OriginPro software was used to generate stress–strain curves and calculate the maximum stress, strain at break, and toughness of the electrospun meshes using the Origin’s integrate function. A standard deviation of all mechanical parameters was calculated from each of the 3 measurements. SEM was utilized to measure the thickness of vertically oriented fibrous samples prepared for mechanical testing, with analysis conducted using ImageJ software. Additionally, SEM was used to verify the fiber morphology after the stretching.

### 2.3. Wetting, Roughness and Fog Collection Experiment

The wetting of meshes was evaluated by the static advancing contact angle measurement (*θ*_adv_). The 3 µL of deionized water (DI, Spring 5UV purification system—Hydrolab, Straszyn, Poland) was applied to the horizontally placed sample (T = 23 °C and RH = 40%). Image of the droplet was taken 3 s after the droplet’s deposition using the camera with a macro lens (EOS 700D, EF-S 60 mm f/2.8 Macro USM, Canon, Tokyo, Japan). The static contact angle was established by assessing images of 10 distinct droplets via ImageJ software and subsequently computing the standard deviation. Similarly, the same method was used to measure the contact angle on vertically positioned meshes. The droplets were deposited during the fog water collection test (FWC), and images were captured before the droplets ran down. The contact angle hysteresis (Δ*θ*_H_) was determined by calculating the difference between the advancing and receding *θ* from 5 droplets.

Surface roughness of the electrospun fiber meshes was analyzed using a laser scanning microscope (Olympus OLS4000, Olympus, Tokyo, Japan). Samples were placed on glass slides for measurement. A consistent scan area of 258 × 258 µm was used for all measurements. For each sample, ten measurements were taken to determine mean values and standard deviations. The surface roughness parameters evaluated were R_a_—average of profile height deviations from the mean line, and R_z_—maximum peak to valley height of the profile within a single sampling length.

The FWC tests were conducted in the environmental chamber presented in Figure S1b in the Supporting Information, where the ultrasonic humidifier (Smart AH900, SETTI+, Łódź, Poland) produced the fog with controlled performance and velocity, reaching 400 mL·h^−1^ and 2 m·s^−1^, respectively. The meshes were cut to size 10 × 10 cm and mounted into the specially designed stand. The membrane thickness was measured using a digital thickness gauge (MarCator 1075 R, Mahr, Göttingen, Germany). Each sample was placed under a 2.9 cm diameter circular stamp, and the gauge’s measuring spindle was gently lowered onto the membrane using the instrument’s nominal contact force (0.5–1 N). For each membrane, measurements were taken at 5 points, and the average value was reported. The fog flow was directed at the mesh at a 90° angle, positioned 6 cm from the output of the humidifier. The distance was adjusted such that the fog flow uniformly covered the entire mesh while minimizing dispersion. The relative humidity in the environmental chamber (50 × 60 × 40 cm) exceeded 90% at a temperature of 23 °C. The stability of the environmental conditions during the process was verified by the thermometer and the hygrometer placed inside the chamber. The water collected by the meshes flowed into a glass beaker placed outside the chamber, which was weighed every 15 min throughout the 2.5 h experiment. Subsequently, the water collection rate was calculated based on the mesh area and a test duration of 1 h. After the FWC experiment, all meshes were weighed to determine the retained water, which was then expressed per unit surface area, similar to a previous study [46].

## 3. Results and Discussion

### 3.1. Material Characterization

**Figure 2:**
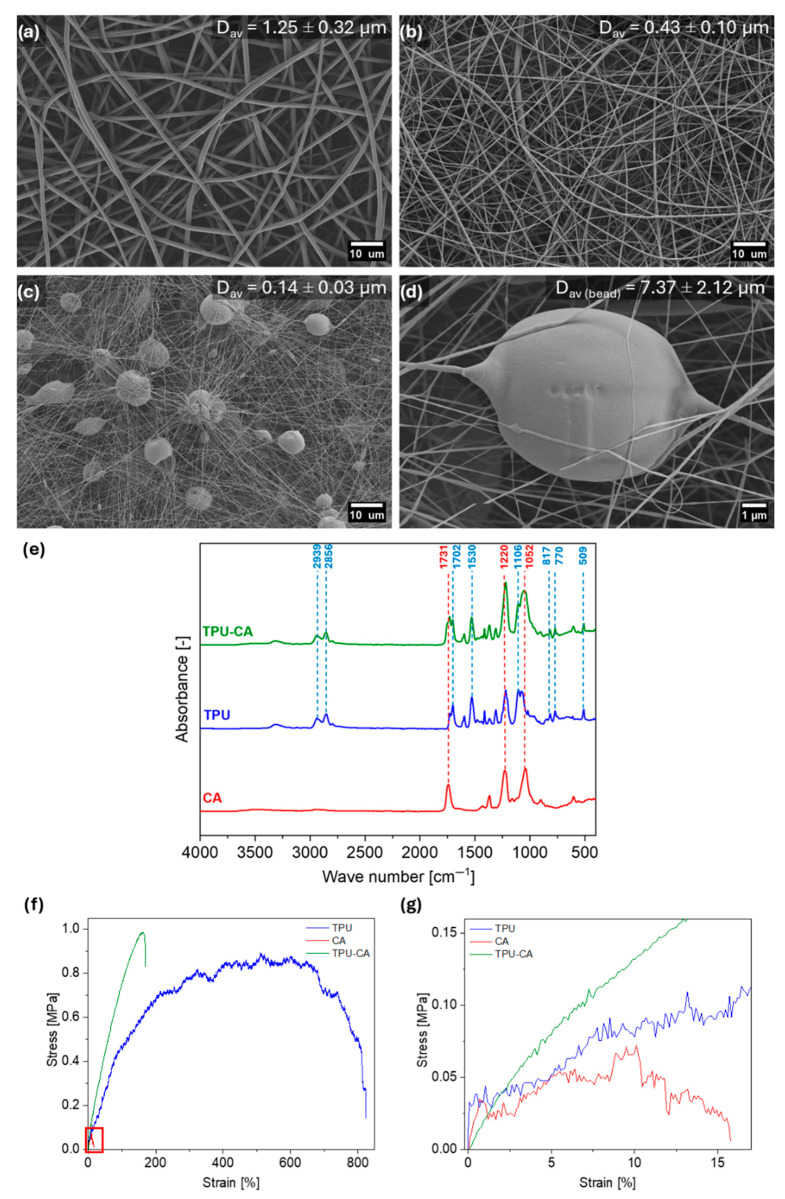
The SEM images of fibers: (**a**) TPU, (**b**) CA, and (**c**) TPU-CA with (**d**) magnification of microbead. (**e**) The FTIR spectra for TPU, CA, and TPU-CA meshes. (**f**) Representative stress–strain curves for TPU, CA, and TPU-CA, and (**g**) the magnification of the CA curve (red frame).

We successfully created flexible fibrous meshes using hydrophobic thermoplastic polyurethane (TPU) and hydrophilic cellulose acetate (CA). The TPU fibers have an average diameter of 1.25 ± 0.32 µm, while the CA fibers average 0.43 ± 0.10 µm. The TPU-CA mesh combines the hydrophobic and hydrophilic properties of both polymers. This structure retains hydrophilic CA microbeads between thin TPU fibers, which have a diameter of 0.13 ± 0.03 µm, approximately 10 times smaller than those of pristine TPU fibers. However, the CA microbeads created on the TPU fibers had average diameters more than 50 times higher than these fibers, which reached 6.68 ± 2.35 µm. The individual meshes were optimized to achieve the most homogeneous fibers. However, developing a new fibrous beaded structure required careful selection of polymer concentration and compatible solvents to maintain a stable cone-jet during co-axial electrospinning. The different combinations of polymer concentration, solvents, and electrospinning parameters used for TPU and TPU-CA resulted in a significant reduction in the diameter of the TPU fibers and the creation of CA microbeads [44,56,57,58]. Reducing the CA solution concentration from 19 wt% to 8 wt% affects viscosity, surface tension, and phase separation behavior, promoting the formation of polymer beads. Microbeads were formed during electrospinning because the polymer solution lacked sufficient viscosity and chain entanglement, resulting in the jet breaking into droplets due to surface tension [59]. Low polymer concentration and high surface tension can destabilize the jet, leading to bead formation instead of smooth fibers. In addition, at such a low polymer concentration, the viscoelastic forces in the CA jet are insufficient to counterbalance surface tension, shifting the process from electrospinning toward an electrospraying-like regime. As a result, the CA solution is unable to form continuous fibers and instead produces discrete microbeads, which is consistent with the well-established critical concentration threshold required for fiber formation. SEM images of the obtained fibers and their size distributions are shown in Figure 2a–d and Figure S2 in the Supporting Information. Using 2D binary SEM images processed with the Li thresholding model, the fractions of hydrophobic TPU fibers and hydrophilic CA beads were quantified based on the distribution of polymer (white) pixels. This analysis yielded a hydrophobic fiber fraction of 73 ± 3% and a hydrophilic bead fraction of 27 ± 4%. As a result of the process, a random distribution of CA microspheres was obtained, ensuring an even presence of hydrophilic regions within the membrane. The CA microspheres enhance wettability and water absorption, while the continuous TPU fiber network provides mechanical stability and enables rapid water removal due to the hydrophobic nature of the fibers. At a CA content of approximately 27%, the spacing between microspheres allows efficient water transport. Consequently, the achieved density and dispersion of CA microspheres ensure a balanced combination of hydrophilic and hydrophobic properties in the membrane.

The chemical composition and the presence of both TPU and CA within the TPU-CA mesh were confirmed using FTIR [60,61,62], see Figure 2e. Stretching vibrations of the C-N bond were observed at 1106 cm^−1^, while bending vibrations of the N-H groups appeared at 1530 cm^−1^. The carbonyl group in TPU also exhibited stretching vibrations at 1702 cm^−1^, a characteristic peak for C=O bonds. Asymmetrical stretching vibrations of CH_2_ groups were noted at 2939 cm^−1^. Peaks were also observed in TPU and CA at a wavenumber of 1731 cm^−1^, likely corresponding to the characteristic C=O stretching bond found in both materials. The analysis also identified CA-specific peaks at wavenumbers of 1220 cm^−1^ and 1052 cm^−1^, corresponding to the stretching vibrations of the C-O acetyl groups and the vibrations within the C-O-C cellulose chain, respectively.

Using the same mixture of solvents for TPU and CA electrospinning solutions enabled the production of electrospun fibers with embedded microbeads, which influenced the mechanical properties of the resulting materials, as illustrated in Figure 2f,g and Figure S3a–c in the Supporting Information. The TPU and TPU-CA meshes exhibited higher mechanical strength than the CA mesh, reaching 0.96 ± 0.13 and 1.02 ± 0.09 MPa, respectively. The TPU-based meshes demonstrated remarkable stretchability [62,63,64], with tensile stress values ten times higher than CA fibers, as summarized in Table S2 in Supporting Information. However, the TPU-CA mesh displayed almost five times lower strain at break compared to pristine TPU, resulting in a corresponding fivefold reduction in the toughness of the fibrous materials. While CA fibers inherently possess significantly lower mechanical properties [44], the reduction in elongation of the TPU-CA mesh is primarily attributed to the geometry of the TPU fibers rather than the incorporation of CA microbeads, see Figure S3d–f in the Supporting Information. However, bead formation on electrospun fibers can compromise their mechanical properties, as the presence of beads disrupts the continuous fiber structure, weakening the material [65]. In our study, this effect is observed in the reduced strain at maximum stress for the TPU-CA fibers compared to the pristine TPU fibers. The SEM images in Figure 2c,d clearly show that beads are not uniformly distributed within the TPU-CA mesh. The microspheres also undergo deformation when the fibers are stretched. The presence of microspheres in electrospun mesh disrupts fiber continuity, introducing structural inhomogeneities that can weaken mechanical properties and create potential breakage points. Thus, the elongation of the samples during the tensile tests is smaller, see Figure 2f. However, beads can also act as crack propagation barriers, contributing to the overall mechanical stability of the mesh. Moreover, coaxial electrospinning with various solutions and process parameters played a key role in achieving a smaller TPU fiber diameter, further affecting the mesh’s strength.

### 3.2. Wetting and FWC Experiments

**Figure 3:**
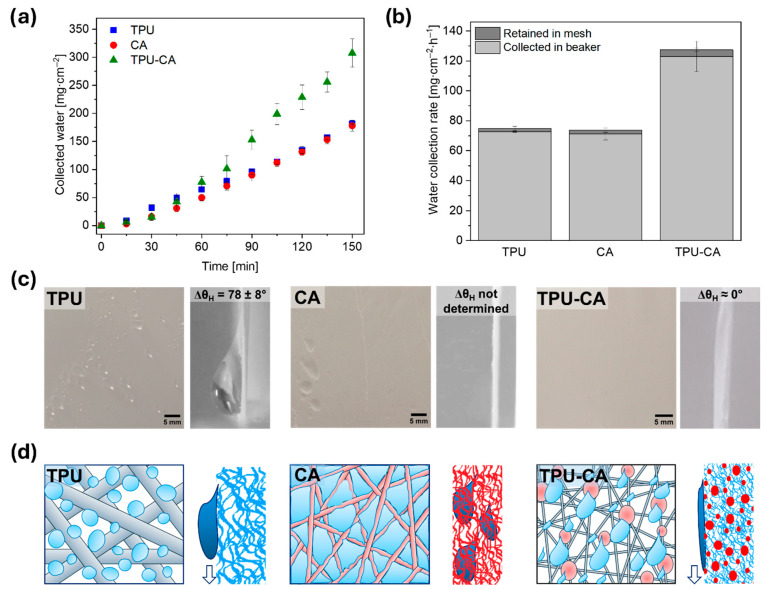
(**a**) The water collected during the FWC test, and (**b**) the water collection rate calculated per 1 h of the experiment. (**c**) The top view of TPU, CA, and TPU-CA meshes after the FWC test and contact angle hysteresis of the flowing droplet. (**d**) The mechanism of catching and running down droplets.

Wetting properties play a crucial role in the development of effective fog collection materials. Environmental factors are independent of us, but recent studies clearly show the advantage of combining hydrophobic and hydrophilic materials [66,67,68,69,70]. The distinct wettability of TPU and CA was confirmed by *θ*_adv_ measurements, as presented in Figure S4 in the Supporting Information. The *θ*_adv_ on the film substrates reached 87 ± 5° for TPU and 56 ± 1° for CA. In contrast, electrospun fibers exhibited a notable increase in *θ*_adv_, reaching 123 ± 4° for TPU and 122 ± 4° for CA, respectively. Comparable values for electrospun fibers have been reported in the literature [71,72]. The observed increase in *θ*_adv_ is attributed to the combined effects of mesh geometry, and surface roughness, consistent with the predictions of the Wenzel and Cassie–Baxter models [73,74,75,76]. Although CA film is intrinsically hydrophilic, the electrospun CA fiber mesh exhibits a high apparent contact angle due to its rough, porous structure. According to the Cassie–Baxter model, air becomes trapped within the fiber network, reducing the solid–liquid contact fraction. This leads to an apparent amplification of hydrophobicity, even though the material itself is hydrophilic. The *θ*_adv_ for hydrophobic TPU fibers with hydrophilic CA microbeads reached 114 ± 4°, confirming the hydrophobicity of the TPU-CA mesh. A similar effect was observed in the previous study, contributing to the mesh’s ability to collect water from fog [44,46]. The literature often emphasizes the high effectiveness of systems based on Janus membranes or cactus spines [77,78,79]. All these structures, which feature distinct hydrophobic and hydrophilic surfaces, enable efficient directional water transport. The hydrophilic side attracts and captures water, while the hydrophobic side facilitates quick transportation, reducing energy loss [80]. This combination significantly enhances water capture efficiency and allows for faster water movement across the surface, improving overall performance in applications such as fog harvesting. Additionally, for materials based on electrospun fibers, an increased accumulation of water droplets on the beads has been observed, driven by the effects of the Laplace pressure, which controls their behavior during accumulation [81,82,83]. However, in this work, we present a different beads-on-surface system, where the effect of Laplace pressure is minimized because hydrophilic microspheres with a high surface area are randomly deposited on the mesh surface rather than forming beads on individual fibers. Notably, the geometry and wetting properties of the TPU-CA mesh enabled the collection of almost twice as much water as the pristine TPU and CA meshes, as shown in Figure 3a,b. The TPU-CA mesh exhibited the highest water collection rate, achieving a total of 127 ± 12 mg∙cm^−2^∙h^−1^, which includes water collected in the beaker and retained within the mesh. The water collection rates of the TPU and CA meshes were lower compared to the TPU-CA mesh, measuring 75 ± 2 and 74 ± 5 mg∙cm^−2^∙h^−1^, respectively. The experiment is conducted for 2.5 h, ensuring that the mesh is fully saturated with water. Figure 3a shows that for individual meshes, the collected water values increase linearly. In contrast, for the TPU-CA, these values start to rise more rapidly after 1 h, suggesting that this is the time required for the mesh to become fully saturated. Only after full saturation does a noticeable difference emerge in the speed at which droplets flow off the meshes. In addition, the electrospun membranes used in this study exhibit comparable thicknesses, ensuring consistent structural conditions across samples: TPU (0.033 ± 0.002 µm), CA (0.023 ± 0.005 µm), and TPU–CA (0.036 ± 0.002 µm). The similarity in thickness further confirms that the differences in fog water collection performance arise primarily from surface wettability and pore-blocking behavior. The proposed architecture is only inspired by key features observed in natural fog-harvesting systems: hybrid wettability and microscale heterogeneity. Introducing hydrophilic domains into an electrospun hydrophobic membrane can enhance water collection while maintaining mechanical stability. Although we did not perform an extensive optimization of all possible geometries and surface patterns, the results clearly demonstrate that the hybrid TPU–CA surface improves droplet nucleation and drainage compared to a purely hydrophobic membrane.

The efficiency of water run-off from the material is indirectly assessed through measurements of contact angle hysteresis (Δ*θ*_H_). The shape of the water droplets, as shown in Figure 3c, clearly illustrates each mesh’s distinct wetting and drainage mechanisms. After fog collection, side-view images of water droplets on the vertical meshes reveal significant variations in Δ*θ*_H_: approximately 78 ± 8° for the hydrophobic TPU mesh and ≈0° for the TPU mesh with hydrophilic microbeads. We observed that the TPU-CA mesh was fully covered with a thin water film, likely due to the increased presence of the hydrophilic fraction in the form of beads scattered across the mesh, see Video S1 in the Supporting Information. However, water drained off from it more quickly than from the other meshes. In contrast, the hydrophilic CA mesh’s tendency to accumulate droplets between the fibers slowed down the water collection process. This pronounced difference in wetting behavior led to varying water removal mechanisms across the measured samples, see Figure 3d. This observation aligns with the theory that a lower Δ*θ*_H_ corresponds to faster droplet run-off [84].

Among the investigated materials, TPU fibers exhibited the lowest surface roughness, with values of R_a_ = 1.59 ± 0.28 µm and R_z_ = 11.31 ± 1.48 µm, see Figure S5 in the Supporting Information. This relatively smooth morphology is advantageous for droplet mobility, as it reduces pinning sites and facilitates water runoff from the surface. The highest roughness was observed for the pure CA mesh, with R_a_ = 5.53 ± 0.61 µm and R_z_ = 29.79 ± 6.10 µm. This may be attributed to electrostatic repulsion between the fibers, resulting in a fluffier mesh structure. The combination of high roughness and the hydrophilic nature of CA promotes water absorption into the porous structure, preventing the measurement of contact angle hysteresis due to continuous wetting and water spreading. The TPU-CA mesh exhibited nearly twice the roughness of neat TPU (R_a_ = 2.42 ± 0.39 µm, R_z_ = 19.36 ± 4.09 µm), primarily due to the presence of CA-derived microspheres distributed across the mesh surface. While this increased roughness could potentially hinder droplet motion, the heterogeneous surface composition and the specific structure of the microspheres appear to enhance droplet dynamics. Moreover, much smaller TPU fibers result in smaller pores and reduce the amount of water clogging the structure. Interestingly, although the TPU-CA composite mesh had higher surface roughness than pure TPU, it exhibited reduced contact angle hysteresis. This behavior likely facilitates faster droplet transport by minimizing pinning forces. As a result, the TPU-CA mesh demonstrated even more efficient droplet flow compared to the pure TPU mesh.

In comparison with our previous studies, this TPU mesh with hydrophilic CA microbeads achieved the highest water collection rate, compared to only 56 mg∙cm^−2^∙h^−1^ for the PS-PA6 micro-nanofiber system and 71 mg∙cm^−2^∙h^−1^ for side-by-side PS-CA [44,46]. In comparison, the pure hydrophobic PVDF mesh achieved a water collection rate of 115 mg∙cm^−2^∙h^−1^, further highlighting the advantages of combining hydrophobic and hydrophilic materials [48]. The improvement in fog collection was moderate. However, the TPU–CA mesh offers important advantages beyond the numerical gain. TPU provides much higher flexibility and mechanical durability than PVDF. Additionally, the inclusion of cellulose acetate, a more environmentally friendly material than fluorinated PVDF, results in a less toxic and more sustainable membrane. Thus, even with a modest performance increase, the TPU–CA system provides clear mechanical and environmental benefits over simple hydrophobic PVDF meshes. In electrospun meshes, the fiber network structure strongly influences their hydrophobicity and directly affects water droplet repellency [85,86]. On the other hand, FWC meshes need to have high fog permeability, with properties analogous to the breathability of materials. High porosity in electrospun nanofiber meshes enhances fog permeability, while rough electrospun surfaces increase hydrophobicity according to the Cassie–Baxter model and effectively limit droplet clusters’ penetration, which reduces pore clogging and improves drainage. Effective fog water collection requires efficient drainage to maintain high permeability, allowing fog to pass freely through the mesh. Electrospun fiber membranes possess a highly permeable structure with around 90% porosity and tunable pore size, as demonstrated in previous studies [87,88,89]. Maintaining this permeability and preventing pore blockage is critical to secure high water collection efficiency. When droplets grow and block the pores, the airflow through the membrane is restricted, significantly reducing the overall performance of the fog water collection process [10,90]. Therefore, hydrophobic or hydrophobic–hydrophilic materials are more commonly utilized, as the hydrophobic component minimizes pore blockage and supports effective drainage [12,91]. The improved fog water collection of the TPU-CA mesh likely results from its unique surface architecture. Our mesh contains randomly distributed hydrophilic CA islands on a hydrophobic TPU background. These hydrophilic domains increase the number of nucleation sites, allowing more droplets to form and be captured. At the same time, the surrounding hydrophobic surface enables fast droplet release, as droplets growing on the hydrophilic islands easily detach and drain due to gravity and the low-adhesion hydrophobic regions. This combination of enhanced droplet nucleation and efficient drainage explains the higher efficiency compared with previously reported materials [92,93]. Direct comparison of fog collection on our meshes and Janus structures is challenging due to the different geometry of the materials and variations in experimental conditions, such as humidifier efficiency, fog flow rate, and sample distance from the source, all of which significantly impact the results [68]. An analysis of various Janus-based systems demonstrates that their water collection rates are significantly higher than those based only on electrospun fibers [94,95,96]. However, their fabrication is significantly more complex and energy-intensive due to laser and chemical treatments, often requiring advanced equipment, which substantially increases production costs. Moreover, these solutions may be size-limited for industrial production, unlike electrospun fibrous systems.

Bead-on-string hierarchical fibers, also created through co-axial electrospinning, were proposed by Thakur et al. [49] as a highly effective solution for fog collection. Their hydrophobic PVDF fibers, combined with hydrophilic PNIPAM beads, successfully mimic the spindle-bead geometry of natural spider silk. The study also demonstrates the importance of environmental conditions in optimizing fog-harvesting systems, highlighting that fog collection efficiency decreased as the chamber temperature increased from 25 to 40 °C due to accelerated droplet evaporation. Hydrophilic beads on the mesh are an excellent alternative to impregnating the mesh with hydrophilic oils. Lalia et al. [84] created electrospun PVDF-HFP nanomats with fiber sizes ranging from 100 nm to 500 nm to capture water from fog and then impregnated them with Krytox-1506 oil to improve the droplet sliding mechanism. This modification allowed them to achieve the water collection rate of 118 mg∙cm^−2^∙h^−1^, which is comparable to the TPU-CA mesh, which has not undergone any chemical modification. Proposed by Ganesh et al. [97], nanofibers with hierarchical structures fabricated using poly (vinylidene fluoride-co-hexafluoropropylene) and fluorinated polyhedral oligomeric silsesquioxane (PVDF-HFP-FPOSS) also showed a lower water collection rate than our fibers, which reached 81 mg∙cm^−2^∙h^−1^.

## 4. Conclusions

The successful fabrication of hydrophobic mesh with hydrophilic microbead made from a combination of TPU and CA using a co-axial nozzle resulted in an enhanced water collection rate compared to pristine meshes applied as fog collectors. The dual hydrophobic–hydrophilic nature of the TPU-CA mesh facilitated an efficient capture and drainage system for the collected water droplets. Incorporating hydrophilic CA microbeads into TPU mesh enhanced the water collection rate of 69%. We achieved a remarkable water collection rate of 127 ± 12 mg∙cm^−2^∙h^−1^. Despite the mesh retaining its hydrophobic nature, water droplets on its vertical surface rolled off completely, exhibiting near-zero contact angle hysteresis. Furthermore, the TPU-CA fibers demonstrated a mechanical strength of 1 MPa, comparable to that of TPU fibers. As a result, the TPU-CA microbead fibers exhibited a significantly improved water collection rate, positioning them as a promising material for water harvesting applications.

## Data Availability

The original contributions presented in this study are included in the article/Supplementary Material. Further inquiries can be directed to the corresponding author.

## Supplementary Materials

The following supporting information can be downloaded at: https://www.mdpi.com/article/10.3390/polym18030425/s1, Figure S1: The schematics of (a) the electrospinning setup with co-axial nozzle, (b) fog water collection setup; Figure S2. The histogram of fiber diameters for electrospun (a) TPU and CA, and (b) TPU-CA, with the histograms of (c) bead diameters. (d–f) The example of TPU-CA SEM image used for binarization and calculation the fiber and beads fraction ratio; Figure S3: Stress–strain curves for: (a) TPU, (b) CA, and (c) TPU-CA; Figure S4: Static contact angles for TPU, CA, and TPU-CA fibers and film; Figure S5: Roughness of the fiber surface: (a) TPU, (b) CA, and (c) TPU-CA, and the graphical explanation of selected parameters: (d) Ra—average of profile height deviations from the mean line, and (e) Rz—maximum peak to valley height of the profile within a single sampling length; Table S1: Electrospinning parameters for produced samples; Table S2: The mechanical properties obtained from stress-strain curves; Video S1: TPU-CA mesh fully covered with a thin water film during fog water collection experiment.
