# Supplementary material for: Hydrophobic Fibers with Hydrophilic Domains for Enhanced Fog Water Harvesting
Source: Polymers (Basel). 2026 Feb 6;18(3):425. doi: 10.3390/polym18030425 (PMC12899429; doi:10.3390/polym18030425)
Supplement: Supplementary file 1 [file polymers-18-00425-s001.zip › polymers-4121274-supplementary.pdf]

Supplementary Material to:

Hydrophobic Fibers with Hydrophilic Domains for Enhanced Fog Water Harvesting

Joanna Knapczyk-Korczak [1], Katarzyna Marszalik [1], Marcin Gajek [2] and Urszula Stachewicz [1,*]

[1] Faculty of Metals Engineering and Industrial Computer Science, AGH University of Krakow, al. A. Mickiewicza 30, 30-059 Krakow, Poland; jknapczyk@agh.edu.pl (J.K.-K.); marszalik@agh.edu.pl (K.M.)

[2] Faculty of Materials Science and Ceramics, AGH University of Krakow, al. A. Mickiewicza 30, 30-059 Krakow, Poland; mgajek@agh.edu.pl

[*] Correspondence: ustachew@agh.edu.pl; Tel.: +48-12-617-44-89

This file includes:

Figure S1. The schematics of (a) the electrospinning setup with co-axial nozzle, (b) fog water collection setup.

Figure S2. The histogram of fiber diameters for electrospun (a) TPU and CA, and (b) TPU-CA, with the histograms of (c) bead diameters. (d-f) The example of TPU-CA SEM image used for binarization and calculation the fiber and beads fraction ratio.

Figure S3. Stress–strain curves for: (a) TPU, (b) CA, and (c) TPU-CA.

Figure S4. Static contact angles for TPU, CA, and TPU-CA fibers and film.

Figure S5. Roughness of the fiber surface: (a) TPU, (b) CA, and (c) TPU-CA, and the graphical explanation of selected parameters: (d) R_a – average of profile height deviations from the mean line, and (e) R_z - maximum peak to valley height of the profile within a single sampling length.

Table S1. Electrospinning parameters for produced samples.

Table S2. The mechanical properties obtained from stress-strain curves.

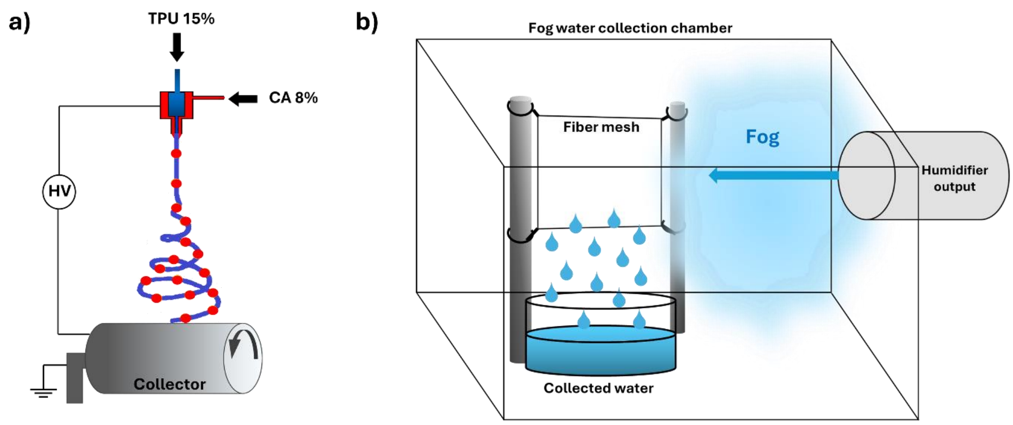

Figure S1. The schematics of (a) the electrospinning setup with co-axial nozzle, (b) fog water collection setup.

Table S1. Electrospinning parameters for produced electrospun meshes from TPU, CA and TPU-CA.

	Electrospinning parameters		
	TPU	CA	TPU-CA
Flow rate [mL·h^{-1}]	1.5	0.1	TPU core = 0.9<br>CA shell = 1.2
Distance [cm]		20	
Applied voltage [kV]	11	16	22
Temperature [°C]	25	25	22
Relative humidity [%]	40	70	50

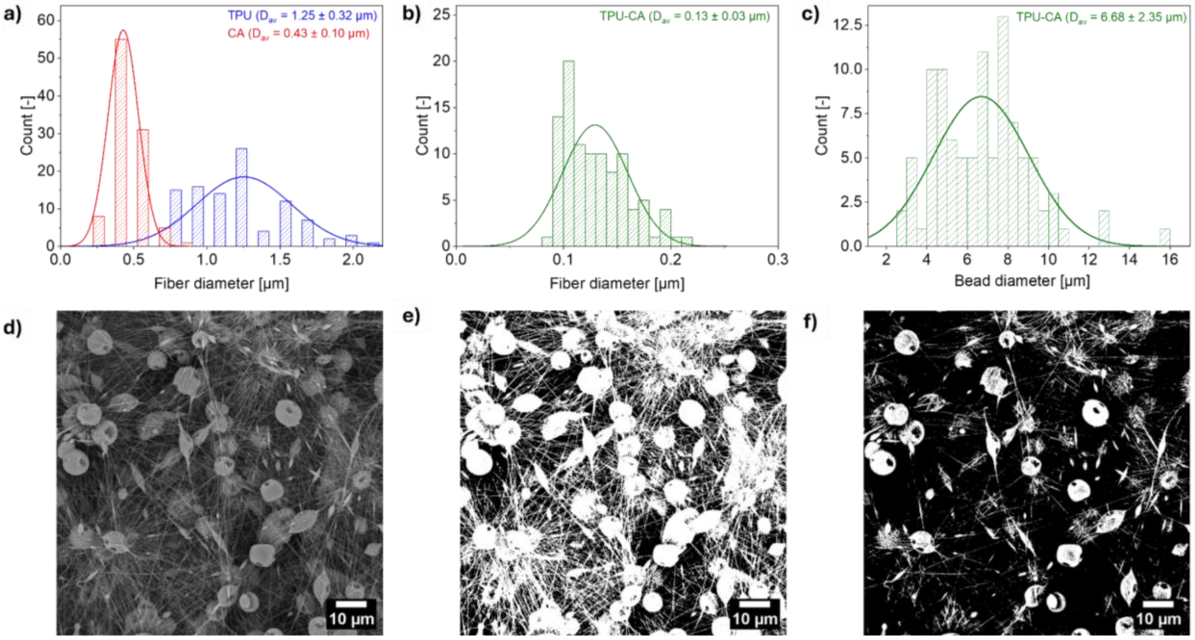

Figure S2. The histogram of fiber diameters for electrospun (a) TPU and CA, and (b) TPU-CA, with the histograms of (c) bead diameters. (d-f) The example of TPU-CA SEM image used for binarization and calculation the fiber and beads fraction ratio.

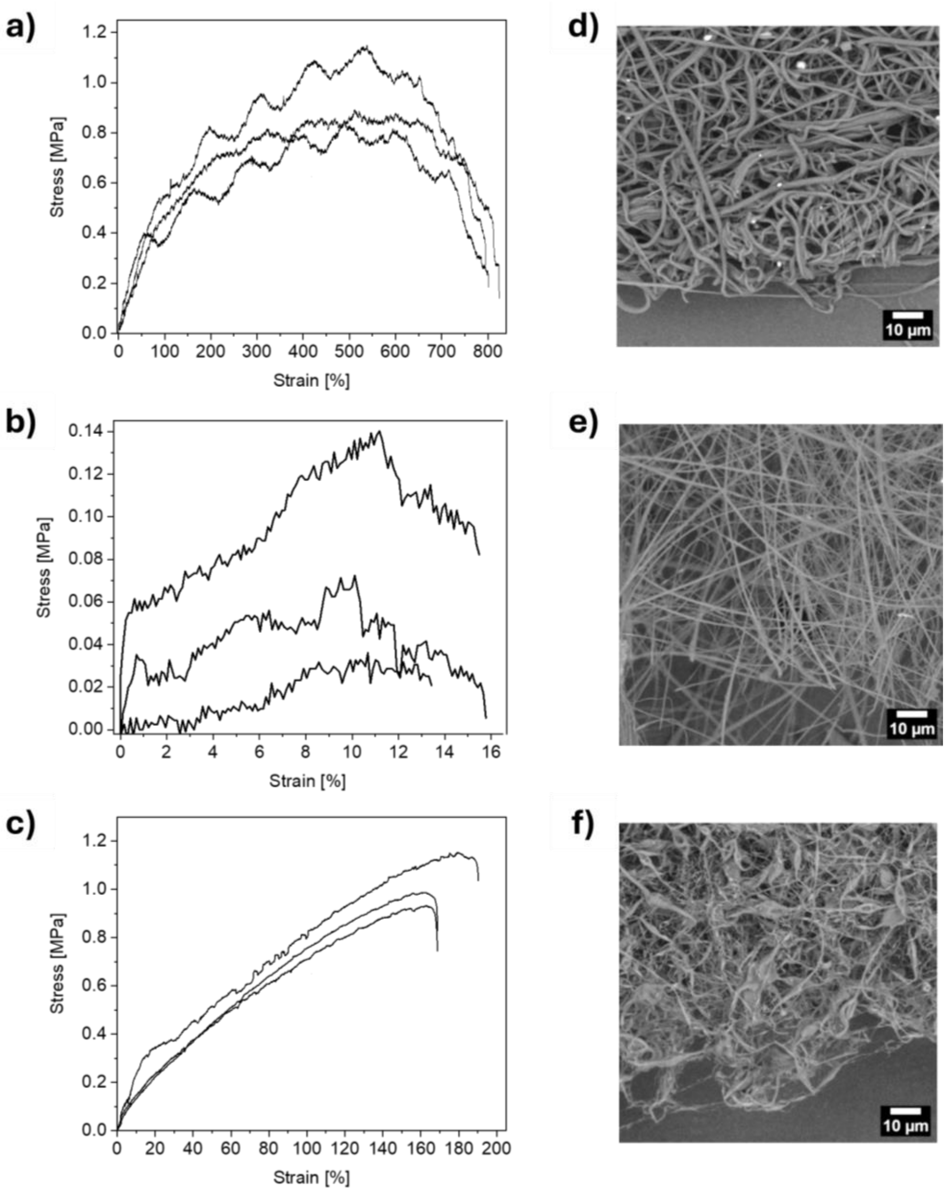

Figure S3. Stress–strain curves for: (a) TPU, (b) CA, and (c) TPU-CA. SEM images of fiber mesh after mechanical testing: (d) TPU, (e) CA, and (f) TPU-CA.

Table S2. The mechanical properties of the electrospun meshes were determined through tensile tests and the resulting stress-strain curves.

	TPU	*Literature data* [1]	CA	*Literature data* [2]	TPU-CA
Maximum stress (MPa)	0.96 ± 0.13	7.8 ± 1.5	0.08 ± 0.04	0.19 ± 0.05	1.02 ± 0.09
Strain at break (%)	822 ± 21	430 ± 140	15 ± 1	167 ± 47	176 ± 10
Toughness ($MJ \cdot m^{-3}$)	5.60 ± 0.68	-	0.01 ± 0.01	16.67 ± 7.26	1.14 ± 0.20

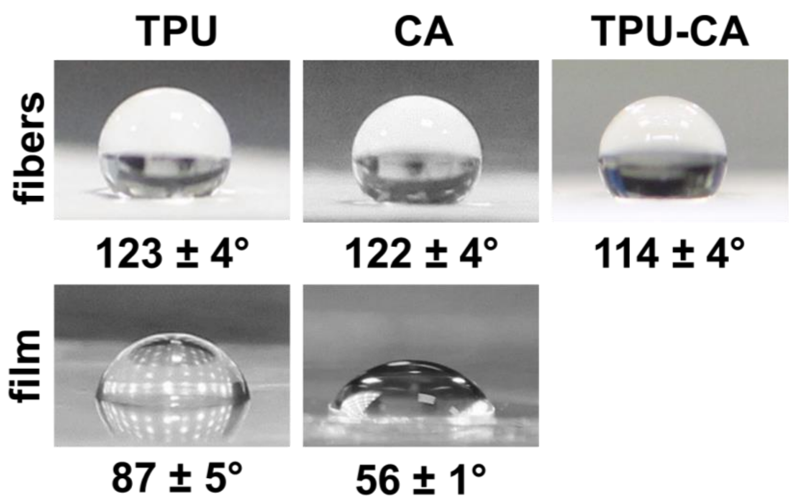

Figure S4. Static contact angles for electrospun fiber mats and film from TPU, CA, and TPU-CA.

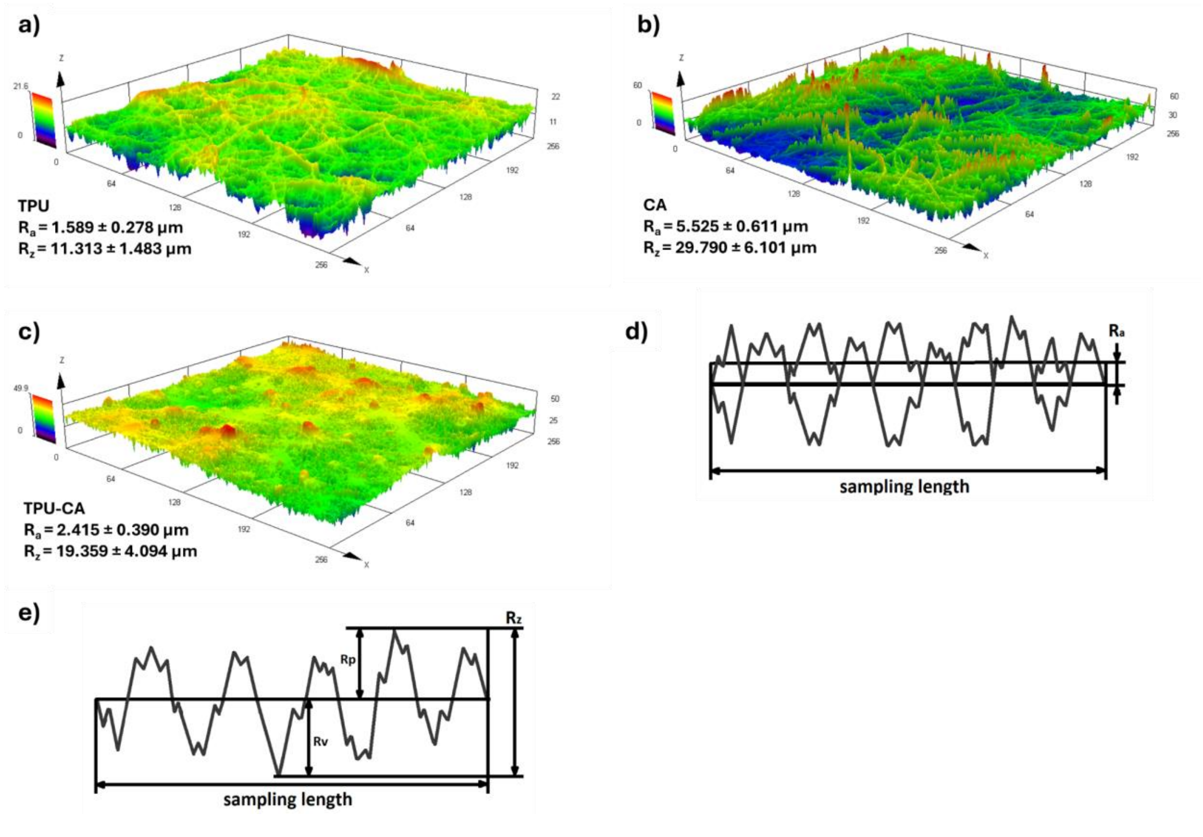

Figure S5. Roughness of the fiber surface: (a) TPU, (b) CA, and (c) TPU-CA, and the graphical explanation of selected parameters: (d) R_a – average of profile height deviations from the mean line, and (e) R_z - maximum peak to valley height of the profile within a single sampling length.

References

(63) Park, C. H.; Kim, C. H.; Tijing, L. D.; Lee, D. H.; Yu, M. H.; Pant, H. R.; Kim, Y.; Kim, C. S. Preparation and Characterization of (Polyurethane/Nylon-6) Nanofiber/(Silicone) Film Composites via Electrospinning and Dip-Coating. *Fibers and Polymers* **2012**, *13* (3), 339–345. https://doi.org/10.1007/s12221-012-0339-x.

(44) Knapczyk-Korczak, J.; Zhu, J.; Ura, D. P.; Szewczyk, P. K.; Gruszczyński, A.; Benker, L.; Agarwal, S.; Stachewicz, U. Enhanced Water Harvesting System and Mechanical Performance from Janus Fibers with Polystyrene and Cellulose Acetate. *ACS Sustain Chem Eng* **2021**, *9* (1), 180–188. https://doi.org/10.1021/acssuschemeng.0c06480.
